# Identification of Gene Regulatory Networks in B-Cell Progenitor Differentiation and Leukemia

**DOI:** 10.3390/genes15080978

**Published:** 2024-07-24

**Authors:** Stefan Nagel, Corinna Meyer

**Affiliations:** Department of Human and Animal Cell Lines, Leibniz-Institute DSMZ, 38124 Braunschweig, Germany

**Keywords:** ETS-code, IRX2, IRX3, NKL-code, TALE-code, TBX-code, TCF3

## Abstract

Pro-B- and pre-B-cells are consecutive entities in early B-cell development, representing cells of origin for B-cell precursor acute lymphoid leukemia (BCP-ALL). Normal B-cell differentiation is critically regulated by specific transcription factors (TFs). Accordingly, TF-encoding genes are frequently deregulated or mutated in BCP-ALL. Recently, we described TF-codes which delineate physiological activities of selected groups of TF-encoding genes in hematopoiesis including B-cell development. Here, we exploited these codes to uncover regulatory connections between particular TFs in pro-B- and pre-B-cells via an analysis of developmental TFs encoded by NKL and TALE homeobox genes and by ETS and T-box genes. Comprehensive expression analyses in BCP-ALL cell lines helped identify validated models to study their mutual regulation in vitro. Knockdown and overexpression experiments and subsequent RNA quantification of TF-encoding genes in selected model cell lines revealed activating, inhibitory or absent connections between nine TFs operating in early B-cell development, including HLX, MSX1, IRX1, MEIS1, ETS2, ERG, SPIB, EOMES, and TBX21. In addition, genomic profiling revealed BCP-ALL subtype-specific copy number alterations of *ERG* at 21q22, while a deletion of the TGFbeta-receptor gene *TGFBR2* at 3p24 resulted in an upregulation of *EOMES*. Finally, we combined the data to uncover gene regulatory networks which control normal differentiation of early B-cells, collectively endorsing more detailed evaluation of BCP-ALL subtypes.

## 1. Introduction

The hematopoietic compartment comprises all developmental entities which generate mature immune and blood cells. The driving processes of hematopoiesis are mainly orchestrated at the transcriptional level and, thus, transcription factors (TFs) form key regulators of hematopoietic development including B-cell differentiation. The human genome contains some 1600 TF-encoding genes, which are classified according to sequence and structure of their DNA-binding domains at the protein level [1,2]. Functionally, some TF groups fill more prominent roles in the regulation of developmental processes, e.g., homeodomain, ETS, and T-box factors. These TFs are frequently mutated and deregulated in developmental diseases and cancer, including leukemia, explaining their prominence in cell and tissue differentiation [3,4,5].

We have established the concept of TF-codes which systematize the physiological activities of key TF groups in developing and mature cell types of the hematopoietic compartment. TF-codes describe gene activities of selected TF-encoding gene groups for each stage of normal development. Thus, they represent a section of specific TFs expressed in a particular lineage of cell differentiation. These codes help to illuminate differentiation processes generating immune and blood cells and serve to unmask aberrant TF expression in corresponding hematopoietic malignancies. So far, our investigations encompass TF-codes for NKL homeobox genes, TALE homeobox genes, ETS genes, and T-box genes [6,7,8,9]. 

TFs orchestrating developmental processes create gene regulatory networks (GRNs). Regulatory connections between particular TFs or even complete GRNs may be highly conserved as described for humans, *Caenorhabditis elegans* and *Drosophila* [10,11]. Exemplary TFs in regulatory relationships driving hematopoiesis include RUNX1 and TCF3 in lymphoid development, TAL1 and GATA3 in T-cell development, and EBF1 and PAX5 in B-cell development [12,13,14,15]. SPIB is an additional fundamental regulator of B-cell development, operating in early stages and final plasma cell differentiation [16]. *SPIB* is regulated by TCF3, PAX5, and RUNX1, demonstrating the highly branched relationships present in B-cell-specific GRNs [17,18,19]. Furthermore, sequencing data generated after chromatin immuno-precipitation have shown that developmental TFs may regulate large numbers of genes, underlining GRN complexity [11].

Deregulation of developmental TFs and their GRNs may underlie the pathogenesis of hematopoietic malignancies by disturbing differentiation processes. For example, TALE homeobox gene *MEIS1* and its target gene *HOXA9* in turn are aberrantly activated by KMT2A fusion proteins in myeloid leukemia and B-cell progenitor acute lymphoid leukemia (BCP-ALL) [20,21]. A reported target gene of HOXA9 is *MEF2C*, both playing fundamental roles in respective hematopoietic stem cell and lymphoid differentiation [22]. *IRX2* and *IRX3* are also oncogenic members of the TALE-class of homeobox genes, deregulating hematopoietic TFs including *TCF3* and *ETV6*, respectively, and activating corresponding oncogenic fusion genes in BCP-ALL [7,23]. 

BCP-ALL is the most frequent malignancy in children [24]. Prognosis and therapy both depend on subtyping the tumor cells. Recently, 23 BCP-ALL subtypes have been identified via cytogenetic and sequencing data [25]. Clinically, the most important characteristics for subtyping remain karyotype and gene fusion, notably *BCR::ABL1*, *ETV6::RUNX1*, *KMT2A::AFF1*, or *TCF3::PBX1* [26]. 

The identification of fused, deregulated, or mutated TFs in BCP-ALL has revealed their physiological function in normal development [15]. Thus, *PBX1*, originally named pre-B-cell homeobox 1, was first isolated as part of the fusion gene *TCF3::PBX1* before its recognition in normal hematopoiesis [27], highlighting combined analysis of normal and aberrantly expressed TFs. We have screened physiological TF activities to evaluate their role in leukemia when deregulated; here, to identify novel and developmentally significant networks, we focus on the TF groups NKL homeodomain, TALE homeodomain, ETS, and T-box, and their roles in normal and malignant B-cells. 

## 2. Materials and Methods

### 2.1. Analysis of Expression Profiling and RNA-Seq Data

Expression data for normal early B-cell types were obtained from Gene Expression Omnibus (GEO, www.ncbi.nlm.nih.gov, accessed on 1 May 2024), using the expression profiling dataset GSE19599 [28], in addition to RNA-seq data from the Human Protein Atlas (www.proteinatlas.org, accessed on 1 May 2024) [29]. For the analysis of cell lines, we exploited RNA-seq data from 100 leukemia/lymphoma cell lines, available at ArrayExpress (www.ebi.ac.uk/arrayexpress, accessed on 1 May 2024) via E-MTAB-7721 [30]. Gene expression profiling data from BCP-ALL patients were examined using datasets GSE79533 and GSE10792 [31,32] and R-based statistical analysis and visualization scripts [33]. 

### 2.2. Cell Lines and Treatments

The cell lines used in this study are held at the DSMZ (Braunschweig, Germany). Information concerning cultivation, classification, and karyotype is given on the website of the cell bank (www.DSMZ.de, accessed on 1 May 2024). All the cell lines had been authenticated (Figure S1) and tested negative for mycoplasma infection. Gene-specific siRNA oligonucleotides were used to modify gene expression levels with reference to AllStars Negative Control siRNA (siCTR) obtained from Qiagen (Hilden, Germany). For overexpression studies, we used commercial cDNAs (IRX1, MEIS1, SPIB) cloned into pCMV6 vectors obtained from Origene (Wiesbaden, Germany). Expression vectors (2 µg) and siRNAs (80 pmol) were transfected into 1 × 10^6^ cells by electroporation using the EPI-2500 impulse generator (Fischer, Heidelberg, Germany) at 350 V for 10 ms. After 20 h of cultivation, electroporated cells were harvested. The cell lines were treated with 100 nM dasatinib (Sigma, Taufkirchen, Germany) or 20 ng/mL TGFb (R&D Systems, Wiesbaden, Germany). The experimental cell line treatments were performed at least twice, generating similar results.

### 2.3. Polymerase Chain Reaction (PCR) Analyses

The TRIzol reagent (Invitrogen, Darmstadt, Germany) or RNeasy Plus extraction kit (Qiagen) were used to extract total RNA from cultivated cells. cDNA was synthesized using 1 µg RNA, random priming, and Superscript II (Invitrogen). A real-time quantitative (RQ) PCR analysis was performed using the 7500 Real-time System and commercial buffer and primer sets (Applied Biosystems/Life Technologies, Darmstadt, Germany). For normalization of expression levels, we quantified the RNA transcripts of the TBP gene. Quantitative analyses were performed as biological replicates and measured in triplicate. Standard deviations are presented in the figures as error bars. Statistical significance was assessed by *t*-tests (two-tailed), and the calculated *p*-values are indicated by asterisks (* *p* < 0.05, ** *p* < 0.01, *** *p* < 0.001, n.s. not significant).

For the detection of fusion transcripts, we performed reverse-transcription (RT) PCR, using oligonucleotides as reported previously [34]. In addition, we analyzed ETV6 and YY1, using the following oligonucleotides: ETV6-for 5′-AGGCCAATTGACAGCAACAC-3′, ETV6-rev 5′-TGCACATTATCCACGGATGG-3′, YY1-for 5′-AAGCAGGTGCAGATCAAGAC-3′, and YY1-rev 5′-CCGAGTTATCCCTGAACATC-3′. All the oligonucleotides were obtained from Eurofins MWG (Ebersberg, Germany). The PCR products were generated using taqpol (Qiagen) and thermocycler TGradient (Biometra, Göttingen, Germany), analyzed by gel electrophoresis and documented with the Azure c200 Gel Imaging System (Azure Biosystems, Dublin, CA, USA). 

### 2.4. Genomic Profiling Analysis

Genomic profiling allows for the comprehensive detection of genomic copy number alterations. The genomic DNA of the BCP-ALL cell lines was prepared with the Qiagen Gentra Puregene Kit (Qiagen). The procedure of labeling, hybridization, and scanning of Cytoscan HD arrays was performed by the Genome Analytics Facility located at the Helmholtz Centre for Infection Research (Braunschweig, Germany), according to the manufacturer’s protocols (Affymetrix, High Wycombe, UK). The associated Chromosome Analysis Suite software version 3.1.0.15 (Affymetrix) was used to generate and illustrate the data.

## 3. Results

### 3.1. TFs in Early B-Cell Development

**Figure 1:**
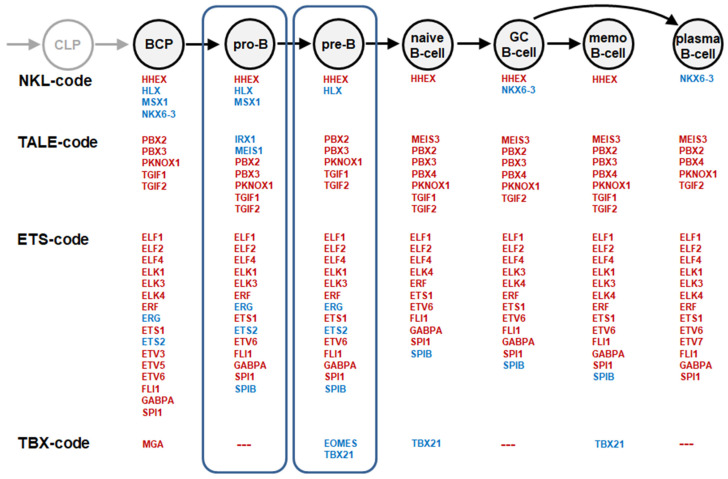
TF-codes in B-cell development. Depicted are the stages of B-cell development (**above**) and the consequently expressed genes of selected TF groups, namely, NKL homeobox, TALE homeobox, ETS, and T-box genes (**below** in red). These TF signatures have been termed NKL-code, TALE-code, ETS-code, and ZBX-code, respectively. TF-encoding genes analyzed in this study are indicated in blue. The pro-B- and pre-B-cell stages and their associated TF activities are framed. Abbreviations: BCP: B-cell progenitor, CLP: common lymphoid progenitor, GC B-cell: germinal center B-cell, memo B-cell: memory B-cell, pre-B: pre-B-cell, pro-B: pro-B-cell.

To describe and evaluate specific TF gene activities in normal and aberrant hematopoiesis, we generated so-called TF-codes [6,7,8,9]. Here, we combined four codes, namely, NKL-, TALE-, ETS- and TBX-codes, and focused on developing and mature B-cell entities. As with our recently reported TALE-code, the remaining three codes were extended by an expression analysis of the pro-B- and pre-B-cell entities [7]. The subsequently generated framework shows TF gene activities at seven consecutive B-cell stages, including four NKL homeobox genes, nine TALE homeobox genes, eighteen ETS genes, and three T-box genes (Figure 1). 

Ascertainment of TF networks may help explain normal B-cell development and predict therapeutic susceptibilities for B-cell malignancies. Accordingly, this study aimed to identify regulatory relationships between TFs operating in normal and aberrant B-cell differentiation. We further focused on pro-B- and pre-B-cell stages which represent the cells of origin for BCP-ALL and are prone for pathogenic TF deregulation [26,35]. We analyzed those TF-encoding genes which physiologically alter their activities within or adjoining the pro-B and pre-B stages, namely, *HLX*, *MSX1*, *NKX6-3*, *IRX1*, *MEIS1*, *ERG*, *ETS2*, *SPIB*, *EOMES*, and *TBX21* (Figure 1). 

**Figure 2:**
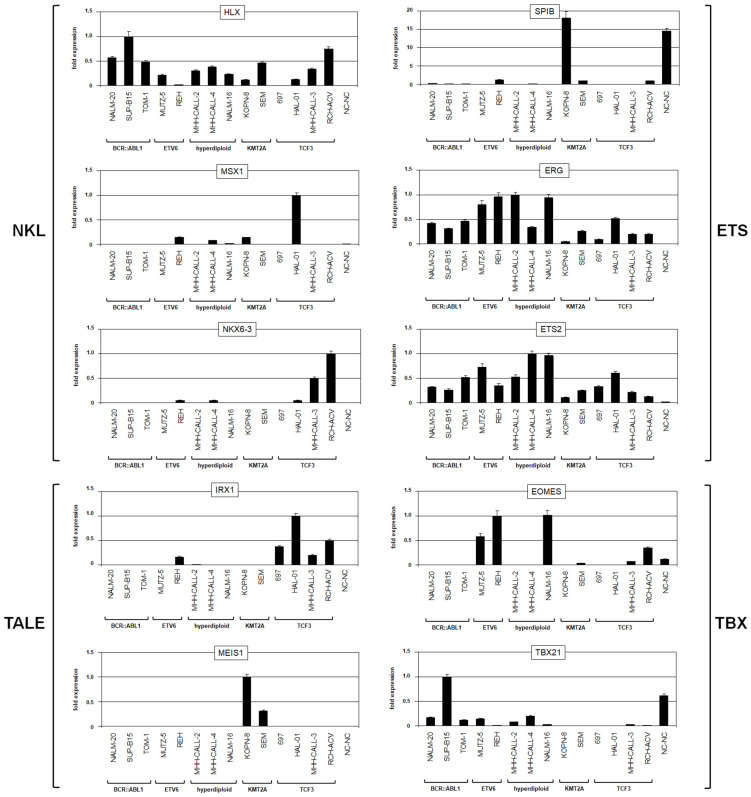
Expression analysis of selected TF-code members in BCP-ALL cell lines via RQ-PCR, quantizing the transcript levels of NKL homeobox genes *HLX*, *MSX1*, and *NKX6-3*, of TALE homeobox genes *IRX1* and *MEIS1*, of ETS genes *SPIB*, *ERG*, and *ETS2*, and of T-box genes *EOMES* and *TBX21*. The cell lines are arranged according to the subtypes BCR-ABL1, ETV6, hyperdiploid, KMT2A, and TCF3. The B-lymphoblastoid cell line NC-NC served as a control corresponding to mature B-cells.

BCP-ALL cell lines are derived from early B-cell entities and mirror specific subtypes, classified by aberrant fusion genes (Figure S2). These cell lines represent optimized models with which the regulation of TF-encoding genes in developing early B-cells can be investigated. Thus, these cells may reveal both normal and abnormal gene regulatory connections. The RQ-PCR analysis of the 10 selected TF-encoding genes revealed cell lines expressing particular combinations of those factors (Figure 2). This information guided the selection of suitable cell line models for analyzing regulatory relationships between B-cell-associated TFs operating in pro-B- and pre-B-cells. Moreover, the data indicated that some of these TF gene activities in BCP-ALL cell lines corresponded to the stage of differentiation arrest they were derived from. Thus, *MEIS1* activity and *TBX21* silencing in KMT2A and TCF3 subtypes, respectively, match the pro-B-cell stage (Figure 1, Figure 2 and Figure S3). However, since other unknown factors may also impact their activities, caution regarding any overly simple explanation for the observed signatures is warranted.

### 3.2. MEIS1, HLX, and MSX1 Are Mutual Regulators in Early B-Cells

**Figure 3:**
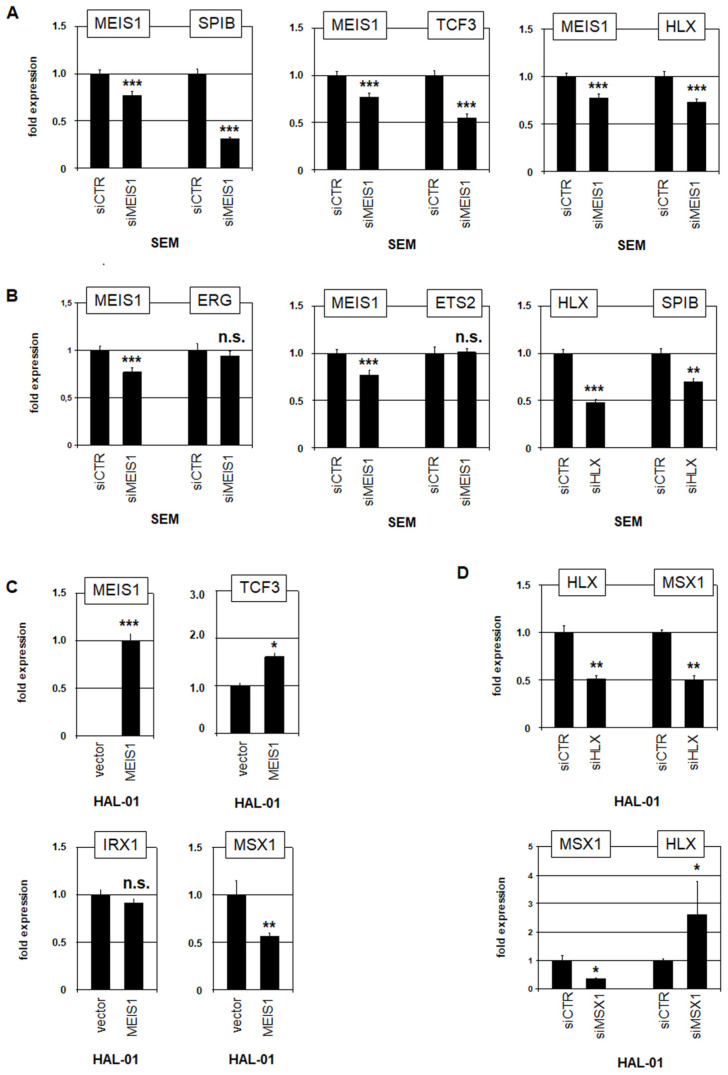
Regulatory activities of MEIS1, HLX, and MSX1. (**A**) SiRNA-mediated knockdown of *MEIS1* in BCP-ALL cell line SEM resulted in reduced expression of *SPIB* (**left**), *TCF3* (**middle**), and *HLX* (**right**). (**B**) SiRNA-mediated knockdown of *MEIS1* in SEM showed no effect on *ERG* (**left**) and *ETS2* (**middle**), while *SPIB* expression decreased (**right**). (**C**) Forced expression of *MEIS1* in *MEIS1*-negative BCP-ALL cell line HAL-01 increased *TCF3* expression (**above**) and showed no effect on *IRX1*, while *MSX1* expression decreased (**below**). (**D**) SiRNA-mediated knockdown of *HLX* (**above**) and *MSX1* (**below**) in HAL-01 resulted in reduced *MSX1* expression and elevated *HLX* expression, respectively. Statistical significance was assessed by *t*-tests (two-tailed), and the calculated *p*-values are indicated by asterisks (* *p* < 0.05, ** *p* < 0.01, *** *p* < 0.001, n.s. not significant).

The TALE-class homeobox gene *MEIS1* is physiologically activated in pro-B-cells and subsequently silenced in pre-B-cells (Figure 1), while KMT2A-rearrangements maintain its aberrant expression [7,20,36]. B-cell line SEM carries the fusion gene *KMT2A::AFF1* (Figure S2) and expresses *MEIS1* in addition to the ETS genes *SPIB*, *ERG* and *ETS2*, and the NKL homeobox gene *HLX* (Figure 2). SiRNA-mediated knockdown of *MEIS1* in SEM resulted in a downregulation of *SPIB* and *HLX*, while *ERG* and *ETS2* activities remained unaltered (Figure 3A,B). *SPIB* is activated by TCF3 as reported previously [17]. Accordingly, our data indicated that MEIS1 may regulate *SPIB* via the activation of *TCF3* (Figure 3A). The knockdown of *HLX* in SEM resulted in a reduced expression of *SPIB* as well (Figure 3B). Thus, *SPIB* is activated by both MEIS1 and HLX, while *ERG* and *ETS2* are not regulated by MEIS1. 

Forced expression of *MEIS1* in BCP-cell line HAL-01 which lacks a *KMT2A*-rearrangement activated *TCF3* (Figure 3C), supporting the data generated in SEM. Furthermore, *MEIS1* overexpression resulted in downregulation of *MSX1*, while *IRX1* remained unaltered (Figure 3C). Knockdown experiments in HAL-01 suppressing *HLX* and *MSX1* demonstrated that HLX activated *MSX1*, while MSX1 inhibited *HLX* (Figure 3D). Collectively, these data show that BCP-ALL cell lines are suitable models for uncovering specific regulatory connections between TFs operating in early-stage B-cells. 

### 3.3. Regulatory Connections between IRX1, SPIB, ERG, HLX, ETS2 and TBX21 in Early B-Cells

**Figure 4:**
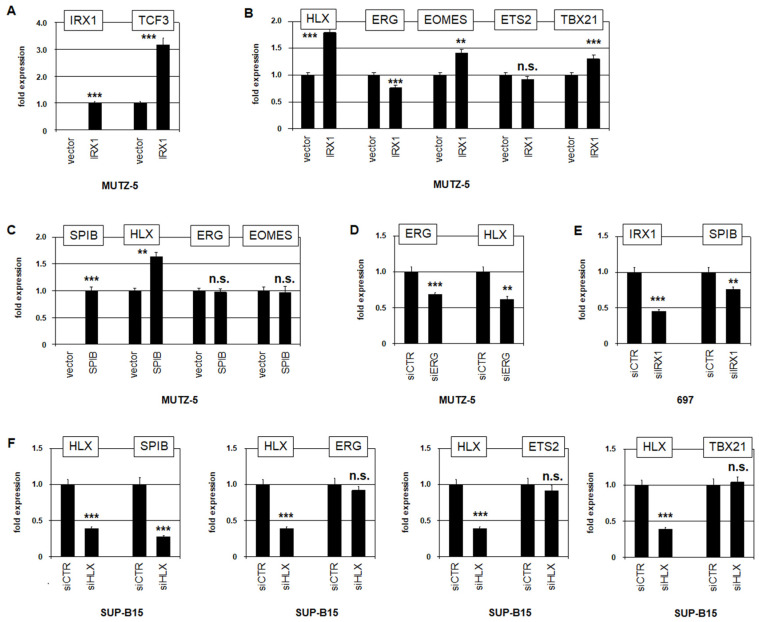
Regulatory activities of IRX1, SPIB, ERG, and HLX. (**A**) Forced expression of *IRX1* in BCP-ALL cell line MUTZ-5 resulted in elevated expression of *TCF3*, supporting reported activity of IRX1 and the effective operation of this assay. (**B**) Forced expression of *IRX1* in MUTZ-5 activated *HLX*, *EOMES*, and *TBX21* and inhibited *ERG*, while *ETS2* expression was not altered. (**C**) Forced expression of *SPIB* in MUTZ-5 resulted in elevated *HLX* expression, while *ERG* and *EOMES* did not alter their expression levels. (**D**) SiRNA-mediated knockdown of *ERG* in MUTZ-5 resulted in reduced expression of *HLX*. (**E**) SiRNA-mediated knockdown of *IRX1* in BCP-ALL cell line 697 resulted in reduced *SPIB* expression. (**F**) In BCP-ALL cell line SUP-B15, siRNA-mediated knockdown of *HLX* resulted in reduced expression of *SPIB*, while the expression levels of *ERG*, *ETS2*, and *TBX21* did not alter significantly. Statistical significance was assessed by *t*-tests (two-tailed), and the calculated *p*-values are indicated by asterisks (** *p* < 0.01, *** *p* < 0.001, n.s. not significant).

The TALE-class homeobox gene *IRX1* shares its restricted activity with *MEIS1* at the pro-B-cell stage in the course of B-cell development (Figure 1). Here, we performed forced expression of *IRX1* in *IRX1*-negative BCP-cell line MUTZ-5. Consistent with a previous report [7], IRX1 activated *TCF3* expression, supporting this approach (Figure 4A). Furthermore, *IRX1* overexpression activated *HLX*, *EOMES*, and *TBX21* and inhibited *ERG*, while *ETS2* remained unaltered (Figure 4B).

ETS-factor SPIB plays an important role in normal B-cell development and in B-cell malignancies when deregulated [37,38,39]. Forced expression of *SPIB* in *SPIB*-negative BCP-cell line MUTZ-5 promoted *HLX* expression while sparing *ERG* and *EOMES* (Figure 4C). Knockdown experiments showed that ERG activated *HLX* in MUTZ-5 (Figure 4D), and IRX1 activated *SPIB* in BCP-cell line 697 (Figure 4E). SiRNA-mediated knockdown of *HLX* in *BCR::ABL1*-positive cell line SUP-B15 resulted in the inhibition of *SPIB*, while *ERG*, *ETS2* and *TBX21* remained unaltered (Figure 4F). Thus, the regulation of *IRX1*, *SPIB*, *ERG*, *HLX*, and *TBX21* is connected in early B-cells. 

### 3.4. ERG Is Targeted by Genomic Aberrations and Fusion Gene BCR::ABL1

**Figure 5:**
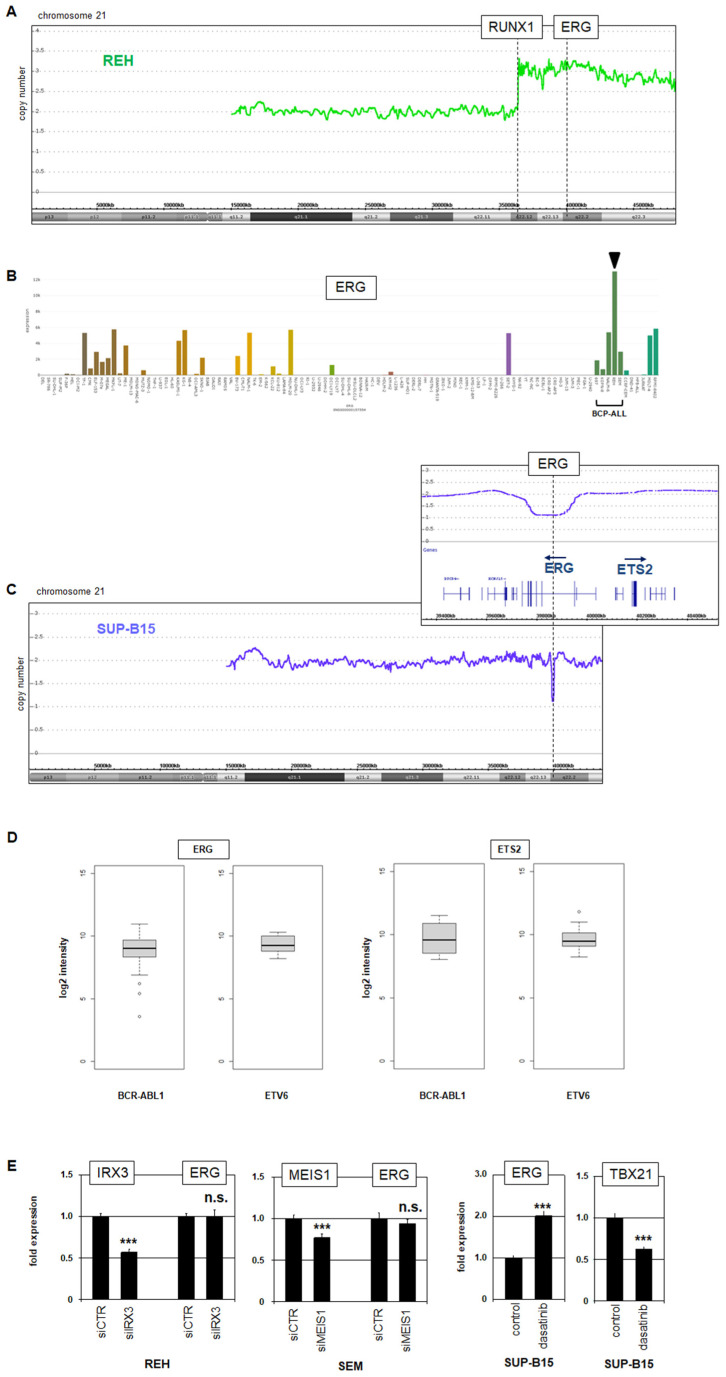
Mechanisms of *ERG* deregulation in B-ALL. (**A**) Genomic profiling data for chromosome 21 from BCP-ALL cell line REH show copy number gain of the *ERG* locus in combination with translocation and fusion of *RUNX1*. (**B**) RNA-seq LL-100 data show enhanced ERG expression levels in BCP-ALL cell line REH (arrow head). BCP-ALL cell lines shown: 697, KOPN-8, NALM-6, REH, and SEM. (**C**) Genomic profiling data for chromosome 21 from BCP-ALL cell line SUP-B15 show focal deletion of the *ERG* locus sparing the neighboring *ETS2* gene. (**D**) Box plots showing expression levels of *ERG* (**left**) and *ETS2* (**right**) from BCP-ALL patients belonging to the respective subtypes BCR-ABL1 and ETV6 (dataset GSE10792). The expression level of *ERG* is significantly lower in BCR-ABL1 patients (*p* = 0.021) while no significant difference was detected for *ETS2*. (**E**) SiRNA-mediated knockdown of *IRX3* (**left**) and *MEIS1* (**middle**) showed no effect on *ERG* expression levels in according BCP-ALL cell lines. Treatment of *BCR::ABL1*-positive cell line SUP-B15 with dasatinib resulted in elevated *ERG* and reduced *TBX21* expression (**right**). Statistical significance was assessed by *t*-tests (two-tailed), and the calculated *p*-values are indicated by asterisks (*** *p* < 0.001, n.s. not significant).

Chromosomal rearrangements play central roles in the leukemogenesis of BCP-ALL. Accordingly, fusion genes are generated by translocations, inversions, or deletions; operate as oncogenes; and serve as biomarkers for subtyping [24]. Fusion gene *ETV6::RUNX1* results from t(12;21)(p13;q22) and is present in BCP-cell line REH (Figure S2) [40]. A copy number analysis of REH showed a gain of the translocated segment 21q22, carrying *RUNX1* in addition to the ETS gene *ERG* (Figure 5A). This genomic duplication corresponded to elevated *ERG* expression in REH as indicated by an RNA-seq data analysis (Figure 4B). Of note, according to CRISPR data from the depmap portal, *ERG* is one of the top-10 preferentially essential genes in REH (depmap.org/portal, accessed on 1 July 2024), supporting an oncogenic role of *ERG* in this cell line. 

In contrast to cell line REH, *BCR::ABL1*-positive BCP-ALL cell line SUP-B15 showed focal deletion of *ERG*, sparing the neighboring *ETS2* gene (Figure 5C). A comparative analysis of BCP-ALL patients using the public dataset GSE10792 demonstrated significantly higher expression levels of *ERG* in *ETV6::RUNX1*-positive than in *BCR::ABL1*-positive patients, while *ETS2* showed no significant difference (Figure 5D), supporting our findings in cell lines. However, knockdown experiments in REH and SEM cells excluded the impacts of the oncogenes *IRX3* and *MEIS1* on *ERG* expression, respectively (Figure 5E). In contrast, treatment of SUP-B15 cells with the ABL1-inhibitor dasatinib resulted in elevated expression levels of *ERG* and a reduction in *TBX21* (Figure 5E). Thus, *ERG* activity distinguishes subtype-specific in BCP-ALL: *ERG* is increased in *ETV6::RUNX1*-positive cases but decreased in *BCR::ABL1*-positive patients. Furthermore, the fusion protein *BCR::ABL1* inhibited the ETS gene *ERG* and activated the T-box gene *TBX21*. 

### 3.5. EOMES Is (De)Regulated by TGFb Signaling

**Figure 6:**
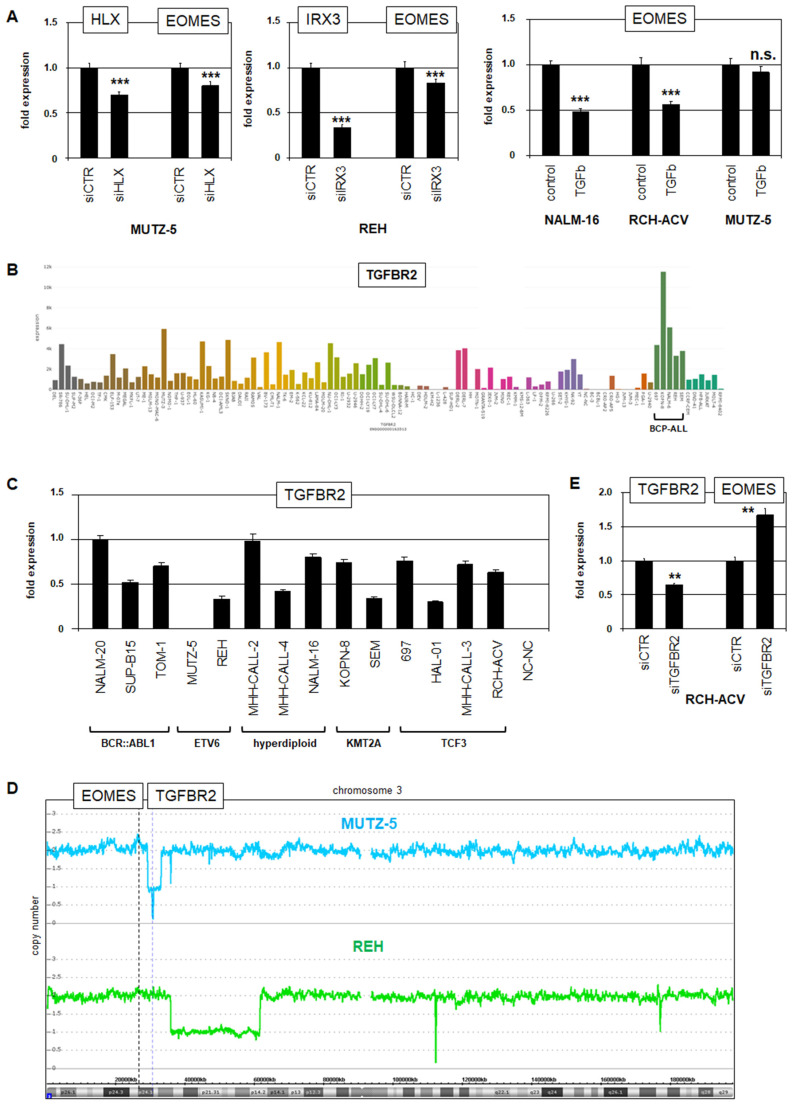
HLX, IRX3, and TGFBR2 signaling regulate *EOMES* in early B-cells. (**A**) SiRNA-mediated knockdown of *HLX* (**left**) and *IRX3* (**middle**) resulted in reduced expression levels of *EOMES*. Treatment of BCP-ALL cell lines NALM-16 and RCH-ACV with TGFb mediated *EOMES* downregulation, while no effect was detectable in MUTZ-5 (**right**). (**B**) RNA-seq LL-100 data show enhanced *TGFBR2* expression levels in BCP-ALL cell lines. BCP-ALL cell lines shown: 697, KOPN-8, NALM-6, REH, and SEM. (**C**) RQ-PCR analysis of *TGFBR2* in selected BCP-ALL cell lines demonstrated absent expression in MUTZ-5. (**D**) Genomic profiling data for chromosome 3 from BCP-ALL cell lines MUTZ-5 and REH show focal deletion of the *TGFBR2* locus in MUTZ-5. (**E**) SiRNA-mediated knockdown of *TGFBR2* in RCH-ACV cells resulted in elevated expression of *EOMES*. Statistical significance was assessed by *t*-tests (two-tailed), and the calculated *p*-values are indicated by asterisks (** *p* < 0.01, *** *p* < 0.001, n.s. not significant).

The expression of T-box gene *EOMES* is restricted to the pre-B-cell stage and has to be silenced for B-cell differentiation to proceed (Figure 1). To analyze the regulation of its specific activity, we used the BCP-ALL cell lines MUTZ-5 and REH (Figure 2). Knockdown experiments indicated that physiologically expressed *HLX* and aberrantly expressed *IRX3* activated *EOMES* expression (Figure 6A). Thus, *HLX* downregulation may promote the silencing of *EOMES* in naïve B-cells, while ectopic expression of *IRX3* may prevent the downregulation of *EOMES* in BCP-ALL. 

Recently, we reported that *IRX3* and the fusion gene *ETV6::RUNX1* are mutual activators [7], while Ford and coworkers have shown that *ETV6::RUNX1* binds and inhibits the TGFb-pathway mediator SMAD3 [41], suggesting regulatory connections. To analyze the potential impact of TGFb signaling on *EOMES* expression, we treated BCP-ALL cell lines with TGFb. In NALM-16 and RCH-ACV, this treatment suppressed *EOMES* expression, but MUTZ-5 showed no effect (Figure 6A), indicating resistance to inhibitory TGFb. 

Using the RNA-seq data of 100 leukemia/lymphoma cell lines, we screened the expression levels of TGFb-pathway members and detected strong activities of the TGFb-receptor gene *TGFBR2* in BCP-ALL cell lines (Figure 6B), which may reflect the physiological significance of this pathway therein. A RQ-PCR analysis of *TGFBR2* in a larger panel of BCP-ALL cell lines confirmed these activities, while in MUTZ-5 and the wild type B-LCL cell line NC-NC, this gene is silenced (Figure 6C). A genomic copy number analysis demonstrated a focal deletion of both *TGFBR2* alleles at chromosomal position 3p24 in MUTZ-5 (Figure 6D), explaining its silencing therein. Of note, the neighboring locus encoding *EOMES* was not affected by this genomic deletion. To investigate the role of *TGFBR2* in EOMES regulation, we performed siRNA-mediated knockdown experiments in RCH-ACV cells. The results demonstrated that TGFBR2 inhibited the expression of *EOMES* (Figure 6E). Taken together, our data show that TGFBR2 signaling supports the suppression of *EOMES* in early B-cells. This regulation is disturbed by the loss of *TGFBR2* or inhibition of SMAD3. 

## 4. Discussion

**Figure 7:**
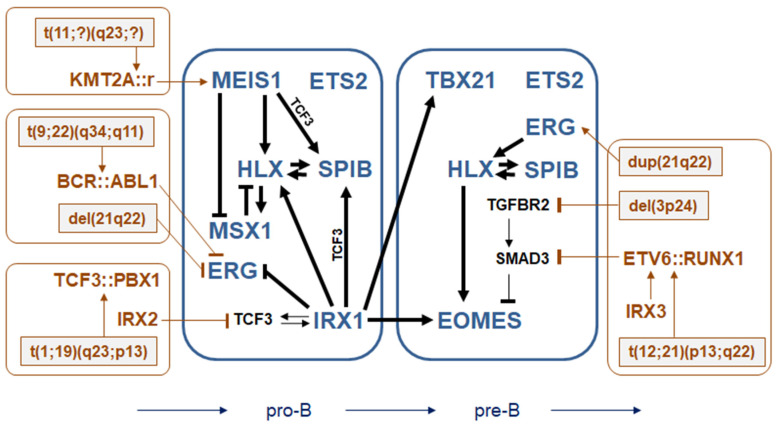
Gene regulatory networks of selected TF-code members (blue) in normal and aberrant early B-cell development as deduced from analyses performed in BCP-ALL cell lines. Additionally, deregulated *IRX* genes, and genomic aberrations generating fusion genes or copy number alterations are indicated (brown).

We have developed the concept of TF-codes to describe TF activities in the course of normal hematopoiesis and to evaluate aberrantly expressed TFs in corresponding malignancies [3,6,7,8,9]. Here, we exploited four TF-codes to uncover regulatory connections between selected TFs expressed in early B-cell development. The results of our study are summarized in Figure 7, showing inferred GRNs in pro-B- and pre-B-cells. We analyzed the activities of developmental TFs encoded by TALE homeobox genes (*IRX1* and *MEIS1*), NKL homeobox genes (*HLX* and *MSX1*), ETS genes (*ERG*, *ETS2*, and *SPIB*), and T-box genes (*EOMES* and *TBX21*), using suitable BCP-ALL-derived cell lines. Additional information concerning fusion genes, copy number alterations, and aberrant *IRX* gene activities is indicated.

The physiological expression of the TALE-class homeobox gene *MEIS1* is restricted to pro-B-cells but is aberrantly activated by KMT2A fusion proteins in BCP-ALL [7,20]. Here, we showed that MEIS1 activated *HLX* and *SPIB* and inhibited *MSX1*. These activities may reflect the physiological regulation by MEIS1 in pro-B-cells. According to the NKL-code scheme, the suppression of *MSX1* is a prerequisite for the progression of normal B-cell differentiation, and reduced *MSX1* expression in BCP-ALL patients is correlated with aberrantly expressed *MEIS1* (Figure S3), supporting the clinical applicability of our findings. 

*IRX1* shares with *MEIS1* its restricted activity to pro-B-cells and the activation of *HLX* and *SPIB*, while IRX1 additionally activated *TBX21* and *EOMES* and repressed the expression of *ERG*. Reportedly, *SPIB* is regulated by TCF3 [17]. Therefore, our data indicated that MEIS1 and IRX1 activated *SPIB* via *TCF3* regulation. Furthermore, IRX1-mediated activation of *EOMES* and *TBX21* may drive the physiological differentiation process, transforming pro-B-cells into pre-B-cells. Collectively, IRX1 shows several regulatory connections to basic TFs, indicating its central role in early B-cell differentiation. 

The ETS gene *ERG* is physiologically silenced after the pre-B-cell stage of early B-cell development and is genomically deleted in subsets of BCP-ALL [42]. We showed that *ERG* is suppressed by IRX1 and that ERG activated *HLX*, as described in NK-cells [43]. Furthermore, we detected an elevated *ERG* expression in correspondence to a copy number gain associated with chromosomal translocation t(12;21)(p13;q22). Elevated *ERG* expression levels have also been reported for *ETV6::RUNX1*-positive patients [44], supporting our findings. In contrast, focal genomic deletion and aberrant BCR::ABL1-signaling in the BCP-ALL cell line SUP-B15 resulted in a premature downregulation of *ERG*. The fusion event resulting in *BCR::ABL1* represents a very early leukemic mutation, while additional gene deregulations, including *ERG* downregulation, may establish a particular group of *BCR::ABL1*-positive BCP-ALL [45]. Thus, the occurrence of untimely *ERG* down- versus upregulation depends on the BCP-ALL subtype. Accordingly, the analysis of BCP-ALL patient data demonstrated that in subsets of *ETV6::RUNX1*-positive cases, *ERG* is upregulated, while subsets of *BCR::ABL1*-positive cases showed *ERG* downregulation (Figure S3). Adding further information to this context, a recent publication by Kodgule and colleagues showed that ERG binds to GGAA microsatellite enhancers and activates according genes in *ETV6*-deficient or *ETV6::RUNX1*-positive tumor cells [46].

The expression of T-box gene *EOMES* is physiologically restricted to pre-B-cells, indicating both a critical function at that particular stage and its required downregulation for progressing B-cell differentiation. Our data demonstrated the activation of *EOMES* by IRX1 and HLX and its repression by TGFb signaling, all of which may represent regulatory mechanisms in normal B-cell development. Interestingly, *EOMES* and *HLX* are both activated by STAT3, constituting an additional connection between these genes [47,48]. Furthermore, we showed that genomic deletion and subsequent silencing of *TGFBR2* resulted in the activation of *EOMES* in *ETV6::RUNX1*-positive cell line REH. These results support recently reported data showing that TGFb-pathway component SMAD3 inhibits *EOMES* and that fusion protein ETV6::RUNX1 binds and inhibits SMAD3 [41,49]. Thus, aberrant activation of *EOMES* may represent an important mechanism of differentiation arrest in *ETV6::RUNX1*-positive BCP-ALL cases. Accordingly, the analysis of BCP-ALL patient data demonstrated that in subsets of *ETV6::RUNX1*-positive cases, *EOMES* is upregulated (Figure S3). However, aberrant expression of *EOMES* was also detectable in subsets of other BCP-ALL subtypes, including BCR::ABL1 and hyperdiploid (Figure S3), indicating the existence of additional deregulatory mechanisms in this malignancy. 

Due to the plethora of possible regulatory connections, we focused on selected TFs. An example of an interesting correlation yet to be further analyzed concerns *NKX6-3*. This NKL homeobox gene is active in B-cell progenitors and silenced in pro-B-cells (Figure 1), suggesting regulatory impacts on *IRX1* and/or *MEIS1*. Furthermore, its aberrant expression in TCF3-subtype BCP-ALL patients and cell lines might also imply an activating role for the fusion gene *TCF3::PBX1* (Figure S3, Figure 2). Thus, our data highlight *NKX6-3* and numerous additional TF activities for investigation in suitable cell line models as prompted by this study. Of note, TF co-expression does not automatically reflect a regulatory connection because the presence of additional unknown players and cofactors, or the absence of TF-binding sites, may significantly impact the expression of the corresponding TFs. 

## 5. Conclusions

The analysis of TF activities in BCP-ALL cell lines as informed by our established TF-codes uncovered regulatory relationships which may play developmental roles in normal pro-B- and pre-B-cells. These findings advance our understanding of differentiation processes in early B-cell development and may help to evaluate subtype-specific therapeutic interventions. Finally, our data should guide the choice of appropriate cell line models for investigating B-cell-associated TFs in vitro. 

## Data Availability

The original contributions presented in the study are included in the article/Supplementary Material, further inquiries can be directed to the corresponding author.

## Supplementary Materials

The following supporting information can be downloaded at https://www.mdpi.com/article/10.3390/genes15080978/s1, Figure S1: STR-profiles of used cell lines; Figure S2: RT-PCR analysis of fusion genes in BCP-ALL cell lines; Figure S3: Expression profiling data from BCP-ALL patients.
