# Supplementary material for: Identification of Gene Regulatory Networks in B-Cell Progenitor Differentiation and Leukemia
Source: Genes (Basel). 2024 Jul 24;15(8):978. doi: 10.3390/genes15080978 (PMC11353346; doi:10.3390/genes15080978)
Supplement: Supplementary file 1 [file genes-15-00978-s001.zip › genes-3075496-supplementary.pdf]

**NALM-20**

| Locus | Allele 1 | Allele 2 |
|---|---|---|
| D5S818 | 11 | 13 |
| D13S317 | 8 | 12 |
| D7S820 | 10 | 12 |
| D16S539 | 9 | 10 |
| vWA | 14 | 18 |
| TH01 | 9 | 9 |
| TPOX | 8 | 11 |
| CSF1PO | 12 | 12 |
| Amelogenin | X | Y |
| D3S1358 | 14 | 15 |
| D21S11 | 30 | 31.2 |
| D18S51 | 16 | 17 |
| PentaE | 9 | 18 |
| PentaD | 11 | 13 |
| D8S1179 | 11 | 16 |
| FGA | 20 | 21 |
| D19S433 | 13 | 14 |
| D2S1338 | 18 | 24 |

**SUP-B15**

| Locus | Allele 1 | Allele 2 |
|---|---|---|
| D5S818 | 12 | 13 |
| D13S317 | 8 | 14 |
| D7S820 | 10 | 11 |
| D16S539 | 11 | 12 |
| vWA | 15 | 17 |
| TH01 | 6 | 9.3 |
| TPOX | 8 | 9 |
| CSF1PO | 11 | 12 |
| Amelogenin | X | Y |
| D3S1358 | 15 | 16 |
| D21S11 | 28 | 31 |
| D18S51 | 14 | 14 |
| PentaE | 21 | 21 |
| PentaD | 9 | 12 |
| D8S1179 | 11 | 14 |
| FGA | 19 | 20 |
| D19S433 | 13 | 15 |
| D2S1338 | 20 | 23 |

**TOM-1**

| Locus | Allele 1 | Allele 2 |
|---|---|---|
| D5S818 | 11 | 13 |
| D13S317 | 8 | 11 |
| D7S820 | 10 | 12 |
| D16S539 | 9 | 10 |
| vWA | 17 | 18 |
| TH01 | 7 | 9 |
| TPOX | 8 | 11 |
| CSF1PO | 10 | 11 |
| Amelogenin | X | X |
| D3S1358 | 16 | 17 |
| D21S11 | 31 | 33.2 |
| D18S51 | 13 | 15 |
| PentaE | 11 | 18 |
| PentaD | 10 | 12 |
| D8S1179 | 13 | 14 |
| FGA | 19 | 23 |
| D19S433 | 14 | 15 |
| D2S1338 | 18 | 18 |

**MUTZ-5**

| Locus | Allele 1 | Allele 2 |
|---|---|---|
| D5S818 | 11 | 11 |
| D13S317 | 10 | 12 |
| D7S820 | 10 | 10 |
| D16S539 | 10 | 10 |
| vWA | 16 | 17 |
| TH01 | 7 | 7 |
| TPOX | 8 | 11 |
| CSF1PO | 8 | 9 |
| Amelogenin | X | X |
| D3S1358 | 15 | 15 |
| D21S11 | 29 | 31.2 |
| D18S51 | 14 | 15 |
| PentaE | 13 | 17 |
| PentaD | 10 | 11 |
| D8S1179 | 10 | 11 |
| FGA | 19 | 23 |
| D19S433 | 14 | 17 |
| D2S1338 | 19 | 21 |

**REH**

| Locus | Allele 1 | Allele 2 | Allele 3 | Allele 4 | Allele 5 |
|---|---|---|---|---|---|
| D5S818 | 11 | 12 | | | |
| D13S317 | 11 | 14 | 10 | | |
| D7S820 | 9 | 12 | | | |
| D16S539 | 9 | 13 | | | |
| vWA | 14 | 15 | 16 | | |
| TH01 | 7 | 9 | | | |
| TPOX | 8 | 8 | | | |
| CSF1PO | 12 | 13 | | | |
| Amelogenin | X | X | | | |
| D3S1358 | 18 | 18 | | | |
| D21S11 | 28 | 31 | 27 | 30 | |
| D18S51 | 10 | 15 | 14 | | |
| PentaE | 7 | 11 | | | |
| PentaD | 10 | 11 | | | |
| D8S1179 | 13 | 15 | | | |
| FGA | 22 | 23 | 13 | | |
| D19S433 | 12 | 14 | | | |
| D2S1338 | 20 | 22 | | | |

**MHH-CALL-2**

| Locus | Allele 1 | Allele 2 |
|---|---|---|
| D5S818 | 12 | 12 |
| D13S317 | 8 | 8 |
| D7S820 | 9 | 9 |
| D16S539 | 14 | 14 |
| vWA | 18 | 18 |
| TH01 | 8 | 8 |
| TPOX | 11 | 11 |
| CSF1PO | 14 | 14 |
| Amelogenin | X | X |
| D3S1358 | 15 | 15 |
| D21S11 | 29 | 30 |
| D18S51 | 12 | 14 |
| PentaE | 12 | 12 |
| PentaD | 9 | 12 |
| D8S1179 | 12 | 12 |
| FGA | 23 | 23 |
| D19S433 | 14 | 14 |
| D2S1338 | 20 | 20 |

**MHH-CALL-4**

| Locus | Allele 1 | Allele 2 |
|---|---|---|
| D5S818 | 13 | 13 |
| D13S317 | 9 | 10 |
| D7S820 | 9 | 11 |
| D16S539 | 11 | 11 |
| vWA | 17 | 17 |
| TH01 | 7 | 9.3 |
| TPOX | 8 | 11 |
| CSF1PO | 11 | 12 |
| Amelogenin | X | Y |
| D3S1358 | 17 | 17 |
| D21S11 | 29 | 31.2 |
| D18S51 | 13 | 15 |
| PentaE | 7 | 12 |
| PentaD | 9 | 13 |
| D8S1179 | 12 | 14 |
| FGA | 21 | 23 |
| D19S433 | 15 | 17 |
| D2S1338 | 17 | 17 |

**NALM-16**

| Locus | Allele 1 | Allele 2 |
|---|---|---|
| D5S818 | 11 | 11 |
| D13S317 | 12 | 12 |
| D7S820 | 10 | 10 |
| D16S539 | 13 | 13 |
| vWA | 17 | 17 |
| TH01 | 8 | 8 |
| TPOX | 11 | 11 |
| CSF1PO | 11 | 11 |
| Amelogenin | X | X |
| D3S1358 | 15 | 17 |
| D21S11 | 29 | 30 |
| D18S51 | 17 | 18 |
| PentaE | 8 | 8 |
| PentaD | 9 | 12.4 |
| D8S1179 | 14 | 14 |
| FGA | 20 | 20 |
| D19S433 | 13 | 13 |
| D2S1338 | 18 | 18 |

**KOPN-8**

| Locus | Allele 1 | Allele 2 |
|---|---|---|
| D5S818 | 9 | 11 |
| D13S317 | 8 | 11 |
| D7S820 | 8 | 10 |
| D16S539 | 9 | 11 |
| vWA | 16 | 18 |
| TH01 | 6 | 10 |
| TPOX | 8 | 11 |
| CSF1PO | 10 | 12 |
| Amelogenin | X | X |
| D3S1358 | 15 | 16 |
| D21S11 | 29 | 29 |
| D18S51 | 14 | 22 |
| PentaE | 12 | 20 |
| PentaD | 12 | 13 |
| D8S1179 | 13 | 16 |
| FGA | 18 | 21 |
| D19S433 | 13 | 14 |
| D2S1338 | 20 | 23 |

**SEM**

| Locus | Allele 1 | Allele 2 | Allele 3 |
|---|---|---|---|
| D5S818 | 12 | 12 | |
| D13S317 | 14 | 14 | |
| D7S820 | 10 | 12 | |
| D16S539 | 8 | 10 | |
| vWA | 17 | 17 | |
| TH01 | 6 | 10 | |
| TPOX | 8 | 8 | |
| CSF1PO | 11 | 12 | |
| Amelogenin | X | X | |
| D3S1358 | 14 | 17 | |
| D21S11 | 29 | 31 | 30 |
| D18S51 | 16 | 17 | |
| PentaE | 10 | 11 | |
| PentaD | 11 | 14 | |
| D8S1179 | 11 | 14 | |
| FGA | 21 | 25 | |
| D19S433 | 13 | 14 | |
| D2S1338 | 17 | 24 | |

**697**

| Locus | Allele 1 | Allele 2 |
|---|---|---|
| D5S818 | 11 | 13 |
| D13S317 | 11 | 12 |
| D7S820 | 10 | 11 |
| D16S539 | 11 | 12 |
| vWA | 16 | 18 |
| TH01 | 8 | 9 |
| TPOX | 8 | 11 |
| CSF1PO | 11 | 12 |
| Amelogenin | X | Y |
| D3S1358 | 16 | 18 |
| D21S11 | 28 | 32.2 |
| D18S51 | 16 | 16 |
| PentaE | 7 | 10 |
| PentaD | 10 | 13 |
| D8S1179 | 13 | 13 |
| FGA | 20 | 23 |
| D19S433 | 12 | 14 |
| D2S1338 | 20 | 21 |

**HAL-01**

| Locus | Allele 1 | Allele 2 |
|---|---|---|
| D5S818 | 12 | 13 |
| D13S317 | 11 | 11 |
| D7S820 | 10 | 12 |
| D16S539 | 9 | 11 |
| vWA | 16 | 17 |
| TH01 | 6 | 8 |
| TPOX | 8 | 11 |
| CSF1PO | 11 | 12 |
| Amelogenin | X | X |
| D3S1358 | 15 | 15 |
| D21S11 | 29 | 29 |
| D18S51 | 13 | 14 |
| PentaE | 11 | 17 |
| PentaD | 9 | 10 |
| D8S1179 | 13 | 15 |
| FGA | 20 | 22 |
| D19S433 | 12 | 14 |
| D2S1338 | 18 | 24 |

**MHH-CALL-3**

| Locus | Allele 1 | Allele 2 |
|---|---|---|
| D5S818 | 11 | 12 |
| D13S317 | 12 | 13 |
| D7S820 | 10 | 12 |
| D16S539 | 11 | 12 |
| vWA | 16 | 16 |
| TH01 | 6 | 8 |
| TPOX | 8 | 10 |
| CSF1PO | 10 | 12 |
| Amelogenin | X | X |
| D3S1358 | 17 | 17 |
| D21S11 | 29 | 29 |
| D18S51 | 17 | 18 |
| PentaE | 7 | 7 |
| PentaD | 9 | 12 |
| D8S1179 | 11 | 13 |
| FGA | 19 | 22 |
| D19S433 | 14 | 15 |
| D2S1338 | 9 | 12 |

**RCH-ACV**

| Locus | Allele 1 | Allele 2 |
|---|---|---|
| D5S818 | 11 | 12 |
| D13S317 | 9 | 14 |
| D7S820 | 10 | 11 |
| D16S539 | 11 | 11 |
| vWA | 16 | 17 |
| TH01 | 6 | 8 |
| TPOX | 8 | 11 |
| CSF1PO | 10 | 11 |
| Amelogenin | X | X |
| D3S1358 | 15 | 15 |
| D21S11 | 29 | 31.2 |
| D18S51 | 13 | 16 |
| PentaE | 7 | 11 |
| PentaD | 9 | 10 |
| D8S1179 | 13 | 15 |
| FGA | 23 | 26 |
| D19S433 | 14 | 14 |
| D2S1338 | 17 | 18 |

**NC-NC**

| Locus | Allele 1 | Allele 2 |
|---|---|---|
| D5S818 | 11 | 11 |
| D13S317 | 12 | 13 |
| D7S820 | 9 | 9 |
| D16S539 | 12 | 13 |
| vWA | 14 | 15 |
| TH01 | 7 | 9.3 |
| TPOX | 8 | 8 |
| CSF1PO | 10 | 12 |
| Amelogenin | X | X |
| D3S1358 | 14 | 18 |
| D21S11 | 29 | 30 |
| D18S51 | 12 | 16 |
| PentaE | 5 | 10 |
| PentaD | 11 | 12 |
| D8S1179 | 13 | 14 |
| FGA | 20 | 24 |
| D19S433 | 14 | 15 |
| D2S1338 | 18 | 22 |

**Fig. S1:** STR-profiles of all cell lines used in this study. STR-profiling was performed at the DSMZ according to standardized protocols.

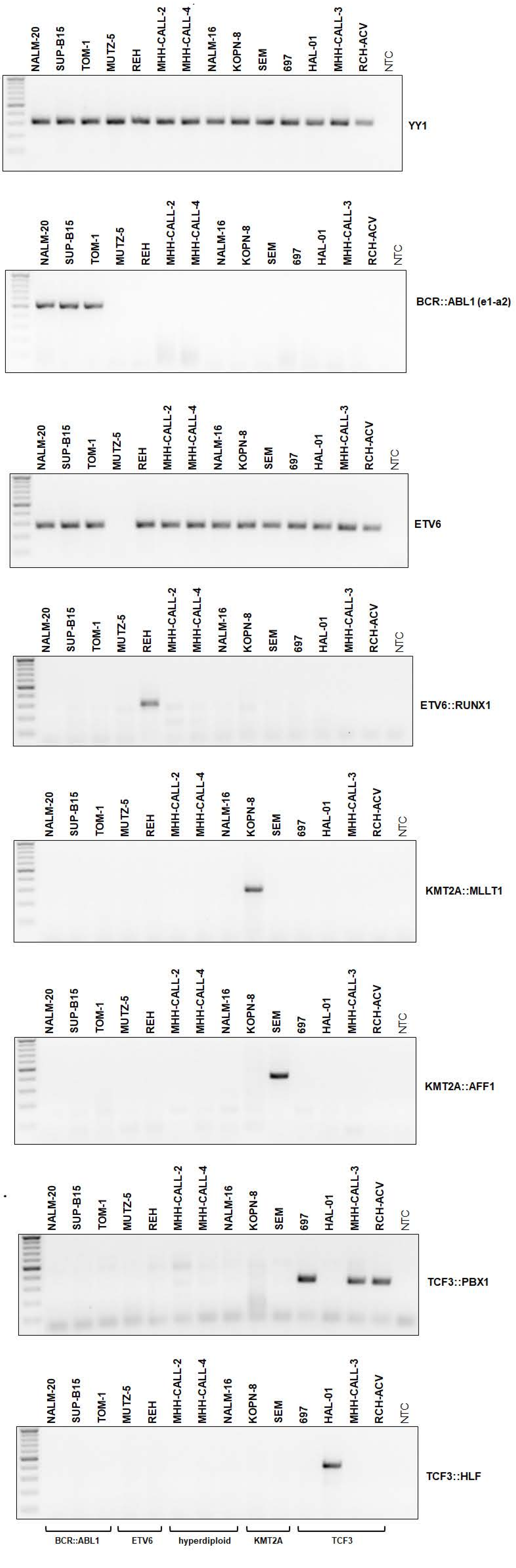


**Fig. S2:** RT-PCR analysis of fusion genes in BCP-ALL cell lines. The cell lines are arranged according to the subtypes BCR::ABL1, ETV6, hyperdiploid, KMT2A and TCF3. Expression of YY1 served as positive control. NTC: no template control.

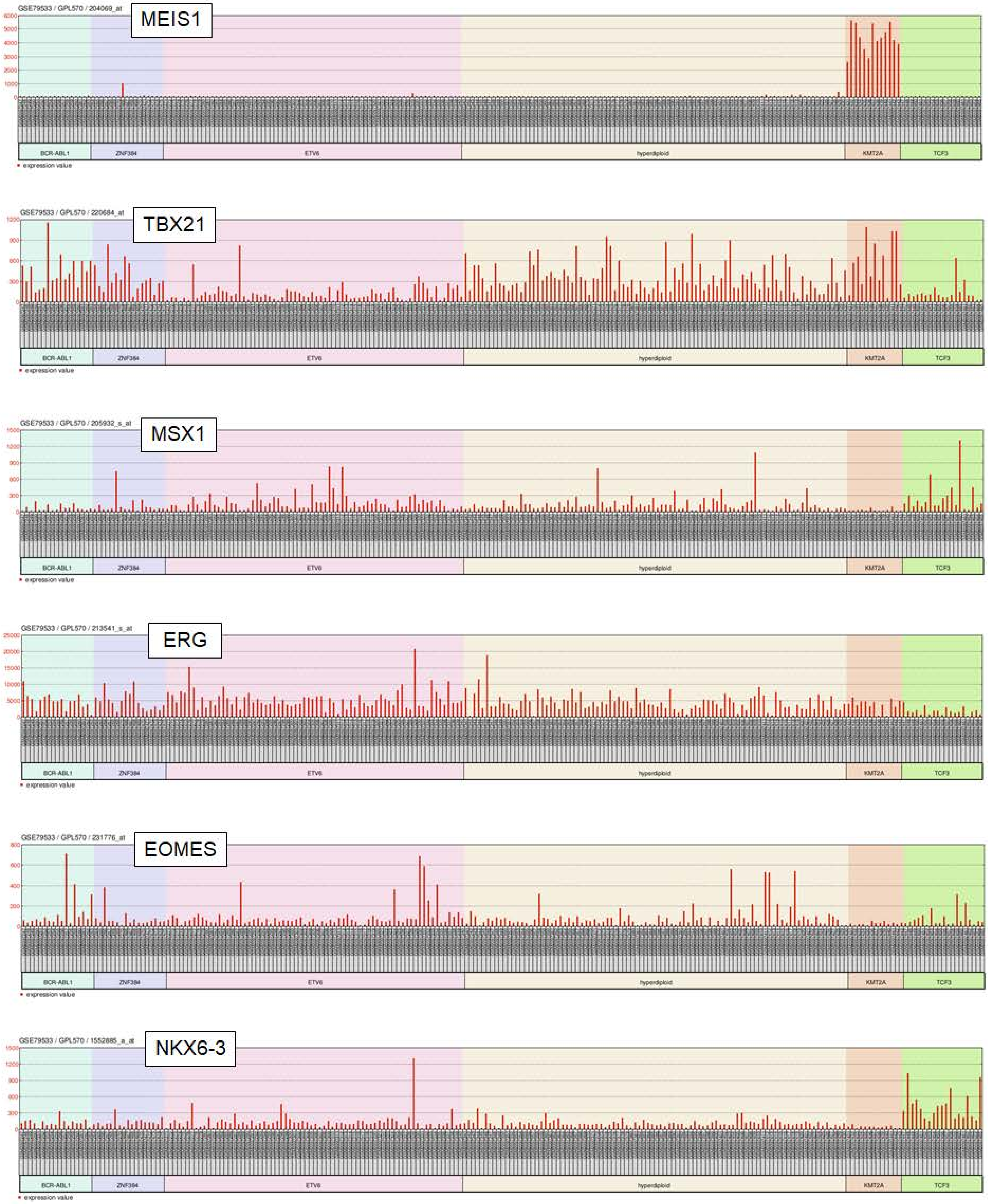


**Fig. S3:** Expression profiling data for MEIS1, TBX21, MSX1, ERG, EOMES and NKX6-3 from BCP-ALL patients (dataset GSE79533). The patients are arranged according to the subtypes BCR::ABL1, ZNF384, ETV6, hyperdiploid, KMT2A and TCF3.
